# Coccidioidomycosis Among Cast and Crew Members at an Outdoor Television Filming Event — California, 2012

**Published:** 2014-04-18

**Authors:** Jason A. Wilken, Patricia Marquez, Dawn Terashita, Jennifer McNary, Gayle Windham, Barbara Materna

**Affiliations:** 1 California Department of Public Health; 2 EIS officer, CDC; 3 Los Angeles County Department of Public Health, Los Angeles, California

In March 2013, the California Department of Public Health (CDPH) identified two Doctor’s First Reports of Occupational Injury or Illness (DFRs)* regarding Los Angeles County residents who had worked at the same jobsite in January 2012 and had been evaluated for possible work-associated coccidioidomycosis (valley fever). Occupational exposure to *Coccidioides*, the causative fungi, typically is associated with soil-disrupting activities (1). The physicians noted that both workers were cast or crew members filming a television series episode, and the site of possible exposure was an outdoor set in Ventura County, California. On the basis of their job titles, neither would have been expected to have been engaged in soil-disrupting activities. Los Angeles County Department of Public Health (LACDPH) conducted an outbreak investigation by using CDPH-provided occupational surveillance records, traditional infectious disease surveillance, and social media searches. This report describes the results of that investigation, which identified a total of five laboratory-confirmed and five probable cases linked to this filming event. The employer and site manager were interviewed. The site manager stated that they would no longer allow soil-disruptive work at the site and would incorporate information about the potential risk for *Coccidioides* exposure onsite into work contracts. Public health professionals, clinicians, and the television and film industry should be aware that employees working outdoors in areas where *Coccidioides* is endemic (e.g., central and southern California), even those not engaged in soil-disruptive work, might be at risk for coccidioidomycosis.

Review of DFRs for coccidioidomycosis diagnoses initially identified two patients who worked for the same employer and listed work-associated coccidioidomycosis as the claimed illness. Patient 1, an actor, sought evaluation at an emergency department on February 28, 2012, after a 2-week history of fever and cough. Patient 1 had received a letter from his employer dated February 17, 2012, stating that a member of the cast or crew present at an outdoor filming event during January 17–19 in Ventura County had a diagnosis of coccidioidomycosis; patient 1 had also worked at this filming event. A copy of the letter sent to the employee was included with the DFR. Patient 2, a camera operator who had sought evaluation at an emergency department on February 24 after a 2-week history of cough, joint aches, and muscle pain, was identified by review of the health-care provider’s notes as having worked at the same outdoor filming event; patient 2 was not the patient referenced in the original letter.

Subsequent review of information obtained from the California Department of Industrial Relations (DIR) identified six additional workers with the same employer who had sought evaluation for possible work-associated coccidioidomycosis. Because all workers identified were residents of Los Angeles County, CDPH informed LACDPH of the possible outbreak, and LACDPH led the local investigation.

A confirmed outbreak case was defined as a laboratory-confirmed illness (including clinical presentation with an influenza-like illness, pneumonia or pulmonary lesion, erythema nodosum or erythema multiforme rash, or extrapulmonary disease) meeting the 2011 Council of State and Territorial Epidemiologists coccidioidomycosis surveillance case definition (3) that occurred in a person who was present at the filming event (performing site preparation work during January 15–16 or at the filming event during January 17–19). A probable case was a clinically compatible illness in a person present at the filming event. Patients were identified through review of DFRs and information obtained from DIR, review of social media, or interview with another patient. LACDPH contacted the employer and obtained cast and crew rosters, which were cross-referenced with the LACDPH coccidioidomycosis surveillance database. Patients, or family contacts of a decedent, were interviewed by LACDPH, and the employer and filming site manager were interviewed by CDPH and LACDPH.

Eight patients initially were identified through review of DFRs and information obtained from DIR. One was identified by review of social media, wherein the patient had posted details about his hospitalization, and one was identified by another patient as a relative (nonemployee) who had been onsite during the filming event. The patient referenced in the employer letter was among those with laboratory-confirmed illness. Of 10 persons identified, seven were interviewed; three could not be contacted. LACDPH ascertained five confirmed and five probable cases. The employee roster indicated 655 workers were associated with that particular television episode. The attack rate for all identified cases was 1.5%.

Median time to symptom onset was 11 days (range = 3–28 days), as determined by interviews of seven patients and medical record review for two patients (Table 1); an estimate could not be made for one patient. Two patients were hospitalized, one for 2 days and one for 4 weeks. The seven interviewed patients reported symptom duration ranging from 1 week to 6 months (Table 2) and reported recovering fully from their illness. One patient had died of an unrelated illness. Five of the interviewed patients reported dry, dusty conditions during the filming event. Only two of the interviewed patients, a construction coordinator and a prop or set maker, engaged in soil-disrupting activities (digging and moving dirt). However, substantial soil-disruptive work, including grading and digging and filling a mud pit, occurred shortly before the filming event. Furthermore, the site manager reported to LACDPH and CDPH that substantial dust from an adjacent mining company blew onto the site daily. CDPH has not identified any cases among employees of the mine at this time.

The employer responded promptly to the initial identification of one illness among cast and crew by sending the original letter to employees, encouraging anyone with symptoms to seek medical evaluation. After interviewing the employer’s environmental health and safety manager and discussing future prevention practices, CDPH provided a “Preventing Work-Related Coccidioidomycosis (Valley Fever)” fact sheet (4) to the employer for integration into their Injury and Illness Prevention Program (IIPP). The site owner informed LACDPH and CDPH that they had already halted digging and excavation at the site. After consultation with CDPH, he stated they would no longer allow soil-disruptive work at the site and would advise future film crews of the potential risk for *Coccidioides* exposure onsite. CDPH also advised the site owner to consult the local air pollution control district for assistance in mitigating offsite dust.

## Discussion

The outbreak described in this report was identified by review of DFRs, using a pilot occupational coccidioidomycosis surveillance system recently established by CDPH. Title 17 of California’s Code of Regulations requires health-care providers to report coccidioidomycosis diagnoses and outbreaks to the local health jurisdiction (5). Although coccidioidomycosis diagnoses for four of the five confirmed cases were reported to LACDPH, the outbreak was only detected by use of a nontraditional database for occupational surveillance. CDPH previously had used workers’ compensation claims data to identify these industries as having the highest incidence of coccidioidomycosis: mining, quarrying, and oil and gas extraction; public administration; agriculture, forestry, fishing, and hunting; and construction (1). Coccidioidomycosis outbreaks among archaeologists (6,7), military personnel (8,9), and construction workers (10) have been described previously. This outbreak investigation identified occupations and an industry not previously known to be at risk.

The outbreak described in this report is illustrative of the risk to employees working outdoors in *Coccidioides*-endemic areas. Although most patients did not engage in soil-disruptive activities, substantial soil disruption immediately preceded the filming event, and the site owner reported ongoing dust intrusion from a neighboring mining company onto the filming site. Because no reliable methods for environmental *Coccidioides* sampling are available, identifying the source of the spores was not possible. CDPH previously had recommended a comprehensive approach to reducing incidence and severity of work-associated coccidioidomycosis (4). The approach includes limiting workers’ exposure to outdoor dust by controlling dust generation at the source (e.g., continuous soil wetting), providing employee training, and consistently enforcing an IIPP, which includes providing respiratory protection with particulate filters. However, the majority of patients in this outbreak were not involved in excavation or set construction and might not have been considered at increased risk for coccidioidomycosis in the existing IIPP. Nevertheless, working at a site immediately after soil disturbance might expose workers to *Coccidioides* spores, and a comprehensive IIPP for these employees should include 1) covering spoils piles and wetting disturbed areas, 2) establishing criteria for suspending work on the basis of wind and dust conditions, and 3) prompt disease recognition and referral to occupational medicine clinics for evaluation, treatment, and follow-up (1,4). Clinicians, including occupational health providers, should be aware that work-associated coccidioidomycosis can occur among patients who do not actively engage in soil-disruptive activities and include relevant information (e.g., employer, worksite, industry, occupation, and other information on activities or locations that might be related to exposure) when reporting cases to local health officials.

What is already known on this topic?

Work-associated *Coccidioides* infections and outbreaks have been linked to soil-disrupting activities, including construction, in areas where *Coccidioides* is endemic.

What is added by this report?

Occupational surveillance identified an outbreak of coccidioidomycosis in an unexpected industry (i.e., film and television). Employees working outdoors in any industry, even those not actively engaged in soil disruption, might be exposed to *Coccidioides* where it is endemic.

What are the implications for public health practice?

Occupational injury and illness surveillance can identify outbreaks not otherwise detected by traditional infectious disease surveillance. Education about coccidioidomycosis, including signs and symptoms, and exposure prevention measures should be implemented at outdoor worksites in areas where *Coccidioides* is endemic, including worksites of industries and occupations not typically associated with soil-disrupting activities. Health-care providers should consider the possibility of work-relatedness among patients with coccidioidomycosis diagnoses and note employer, work location, industry, and occupation when reporting cases.

## Figures and Tables

**TABLE 1:** Demographic characteristics and outcomes of coccidioidomycosis patients — California, 2012

Characteristic	No.
**Case status**	
Confirmed	5
Probable	5
**Age (yrs) median (range)**	37 (23–58)
**Male**	7
**Race**	
White	6
Black	3
Asian	1
**Visited emergency department**	5
**Hospitalized (2–28 days)**	2
**Time to symptom onset (days) median (range) (9 patients)**	11 (3–28)

**TABLE 2:** Occupation and outcomes of coccidioidomycosis patients — California, 2012

Patient no.	Confirmed/Probable	Interviewed	Occupation	Time to illness onset (days)	Hospitalized	Symptom duration	Identification source
1	Probable	No	Actor	28	N/A	N/A	DFR
2	Probable	No*	Sound technician	15	N/A	N/A	DFR
3	Confirmed	Yes	Prop/Set construction	4	No	4 wks	DIR
4	Confirmed	Yes	Actor	6	No	1 wk	DIR
5	Probable	Yes	Actor	3	No	3 wks	DIR
6	Probable	No	Actor	N/A	N/A	N/A	DIR
7	Confirmed	Yes	Camera operator	22	No	6 mos	DIR
8	Probable	Yes	Construction manager	11	No	3 wks	DIR
9	Confirmed	Yes	Actor	7	4 wks	4 wks	Social media
10	Confirmed	Yes	N/A (visitor)	15	2 days	3 wks	Patient interview

**Abbreviations:** DFR = Doctor’s First Report of Occupational Injury or Illness; DIR = California Department of Industrial Relations; N/A = not available.

* Deceased from unrelated illness; family contacts interviewed.

## Footnotes

* In California, health-care providers who believe a patient’s injury or illness might be work-related are required to submit a DFR to the employer or their workers’ compensation insurance carrier, who forward it to the California Department of Industrial Relations (2). DFRs are provided to CDPH for occupational injury and disease surveillance purposes.
